# Suppression of the growth and metastasis of mouse melanoma by *Taenia crassiceps* and *Mesocestoides corti* tapeworms

**DOI:** 10.3389/fimmu.2024.1376907

**Published:** 2024-03-20

**Authors:** Manfred Schreiber, Tomáš Macháček, Vojtěch Vajs, Barbora Šmídová, Martin Majer, Jiří Hrdý, Ondřej Tolde, Jan Brábek, Daniel Rösel, Petr Horák

**Affiliations:** ^1^ Department of Parasitology, Faculty of Science, Charles University, Prague, Czechia; ^2^ Institute of Immunology and Microbiology, First Faculty of Medicine, Charles University and General University Hospital, Prague, Czechia; ^3^ Department of Cell Biology, and Biotechnology and Biomedicine Center of the Academy of Sciences and Charles University in Vestec (BIOCEV), Charles University, Prague, Czechia

**Keywords:** melanoma, *Taenia*, *Mesocestoides*, cancer, suppression, tapeworm, metastasis

## Abstract

Cancer is still one of the leading causes of death, with an estimated 19.3 million new cases every year. Our paper presents the tumor-suppressing effect of *Taenia crassiceps* and *Mesocestoides corti* on B16F10 melanoma, the intraperitoneal application of which followed the experimental infection with these tapeworms, resulting in varying degrees of effectiveness in two strains of mice. In the case of *M. corti*-infected ICR mice, a strong tumor growth suppression occurred, which was accompanied by a significant reduction in the formation of distant metastases in the liver and lung. Tapeworm-infected C57BL/6J mice also showed a suppression of tumor growth and, in addition, the overall survival of infected C57BL/6J mice was significantly improved. Experiments with potential cross-reaction of melanoma and tapeworm antigens with respective specific antibodies, restimulation of spleen T cells, or the direct effect of tapeworm excretory-secretory products on melanoma cells *in vitro* could not explain the phenomenon. However, infections with *T. crassiceps* and *M. corti* increased the number of leukocytes possibly involved in anti-tumor immunity in the peritoneal cavity of both ICR and C57BL/6J mice. This study unveils the complex interplay between tapeworm infections, immune responses, and melanoma progression, emphasizing the need for further exploration of the mechanisms driving observed tumor-suppressive effects.

## 1 Introduction

Melanoma is a cancer stemming from malignant melanin-producing cells – melanocytes (1). It primarily occurs in the skin but may also originate in the eyes, the gastrointestinal tract, or oral, genital, and nasal mucous membranes (2). The incidence of this cancer has expanded in developed countries, such as the US and the UK (3). Melanoma accounts for 80% of skin cancer deaths (1), and it has been estimated that 57,000 have died of melanoma in 2020 (4). It is at the forefront of cancer diagnosis in the developed world, and projections point towards an increase in incidence in the coming decades. For patients diagnosed with stage IV of the disease, the survival rate is dismal, though overall mortality rates have decreased due to advances in targeted- and immuno-therapies (3).

Research focusing on the interactions between certain parasitic organisms and cancer has emerged in recent years. While some species of helminths are known to be the causative agents of cancer, such as *Opisthorchis felineus* (5) and *Schistosoma haematobium* (6), there are some examples of helminths which may have the opposite effect. Indeed, the infection with *Echinococcus granulosus* has already shown the ability to protect against breast cancer in a rat model (7). Moreover, a Kunitz-type protease inhibitor from the same tapeworm induced apoptosis of several cancer cells while not affecting healthy ones (8). The immunomodulatory excretory-secretory products of *Angiostrongylus cantonensis* were shown to significantly reduce tumor growth in a mouse model of hepatocarcinoma as well as negatively affect human hepatocarcinoma cells *in vitro* while having no inhibitory effect on healthy liver cells (9). The infection with *Trichinella spiralis* can then reduce solid tumor burden in a B16F10 mouse model (10). Overall, cases of cancer suppression by helminths or their products exist, but the mechanisms are still largely speculative and unknown.

*Taenia crassiceps*, a tapeworm from the order Cyclophyllidea, which is a parasite of carnivores, such as foxes and wolves, is usually found in its intermediate host (various small rodents) subcutaneously, intraperitoneally, or intrapleurally in the form of cysticerci, which readily grow and asexually multiply. This ability and the relative ease of maintaining a laboratory strain through intraperitoneal passage and the fact that it does not readily infect humans make it an ideal model organism (11). *T. crassiceps* can affect the host at the physiological and immunological levels. It can feminize a male intermediate host (12), but more importantly, it modulates the host’s immune response. While the primary immune response to *T. crassiceps* infection is represented by a pro-inflammatory Th1 type, a chronic anti-inflammatory Th2 is established during the infection. This effect depends upon the parasite’s excretory-secretory products (13), which can elicit the effect on their own (14). What is paramount, however, is that the infection with *T. crassiceps* inhibited tumor formation in colitis-associated colon cancer in a mouse model (15), while said excretory-secretory products were able to do the same by directly affecting the cancer cells (16). Furthermore, GK-1, a synthetic peptide with adjuvant properties originally isolated from this tapeworm (17), has been successfully used in combination with the highly immunogenic MAGE-AX peptide to increase apoptosis and necrosis of melanoma tumors in a mouse model (18).

Similarly, *Mesocestoides corti* (syn. *M. vogae*), a tapeworm of the same order, which infects carnivores through a cycle encompassing various intermediate hosts (mice, birds, amphibians, or reptiles), develops into a tetrathyridium which is also capable of extensive asexual multiplication within the body cavity of an experimental host, giving much the same reasons for being an optimal laboratory model as *T. crassiceps* (19). In contrast, *M. corti* tetrathyridia penetrate the liver parenchyma (20), causing fibrosis (21). The first response of the host immune system to an *M. corti* infection is also of the Th1 type (22). In the first two weeks after infection, there is an increase in CD8+ T cells and NK cells. The tapeworm later suppresses these activities, interrupting the IFN-γ/IL-4 signaling pathways and switching the immune response to an anti-inflammatory Th2 conducive to parasite survival. The tapeworm’s excretory-secretory products themselves are capable of inducing this effect upon injection into the peritoneal cavity (23).

The B16F10 melanoma cell line is a highly aggressive pigmented variant of the B16 line, originally isolated from the C57BL/6 mouse. It may metastasize into the lungs (24) as well as other organs and areas, such as bones and various soft tissues (25) and lymph nodes (26). It is usually introduced intravenously or subcutaneously but may also be injected intraperitoneally (27). In the latter case, disseminated abdominal metastases form (28).

While helminth parasites might have a potential role in the fight against cancer, it is still important to note that they are pathogenic organisms and can cause harm to the host when introduced whole, especially if patients with oncological afflictions are to be considered. Besides the study of the cancer-suppressing effect as a whole, it is necessary to identify the underlying mechanisms of the phenomenon, as well as the components of the tapeworm or their excretory-secretory products, which is the focus of this study. In particular, we studied the suppressive effect of the *T. crassiceps* and *M. corti* infections on the growth and metastasis of the B16F10 melanoma cell line in the mouse model and the possible pathways through which it can affect either the melanoma cells or the host immune system, specifically looking at the humoral and cellular immune response and the effect of parasites’ excretory-secretory products on the cancer cell line.

## 2 Materials and methods

### 2.1 Ethics statement

All experiments were performed in accordance with the animal welfare laws of the Czech Republic and were approved by the Animal Welfare Committee of Charles University, Faculty of Science (project ID: MSMT-33097/2020-6). All authors of this paper are authorized to design and perform experiments with animals. Consent to participate is not applicable.

### 2.2 Mice

The C57BL/6JOIaHsd inbred strain was acquired from Envigo, and the Hsd : ICR (CD-1^®^) outbred strain was acquired from Charles River Laboratories (females aged 5 weeks in all cases). The mice were kept at the Centre for Experimental Biomodels (1st Faculty of Medicine, Charles University). They were housed in conventional cages with a 12h-12h light cycle at 23°C with 55% air humidity and *ad libitum* access to water and feed.

### 2.3 Parasites

The *Taenia crassiceps* isolate (originally from Janssen Pharmaceutica, Beers) was provided by Prof. Pierre Dorny (Institute of Tropical Medicine, Antwerpen, Belgium). The original host organism is unknown. The isolate has been kept in a laboratory for over 15 years. The *M. corti* isolate was acquired from Dr. Ruth Fichter and Prof. Peter Deplazes (Institute of Parasitology, University of Zürich, Switzerland). The isolate was originally acquired from an infected squirrel (*Sciurus vulgaris*) from Wedmark, Germany. The larval stages of *T. crassiceps* and *M. corti* were harvested from the peritoneal cavity of female ICR mice 3 months post infection (p.i.). They were then washed five times with sterile PBS and injected intraperitoneally. The infection doses for *T. crassiceps* and *M. corti* were 30 cysticerci and 600 tetrathyridia, respectively.

### 2.4 Design of *in vivo* experiments

Mouse survival: Groups of 10 five-week-old C57BL/6J females were infected with *T. crassiceps* or *M. corti* or injected with sterile PBS. After 2 weeks, when the tapeworm larvae have established an infection, 5×10^5^ B16F10 melanoma cells were injected into their peritoneal cavity, upon which they were observed throughout 26 days.

Tumor growth suppression, histological and immunological evaluation: Groups of 7 five-week-old C57BL/6J or ICR female mice were injected intraperitoneally with *T. crassiceps* or *M. corti* larvae. In order to ensure longer mouse survival for immunological evaluation, a smaller dose of 3×10^5^ B16F10 melanoma cells suspended in sterile PBS was introduced after two weeks into the peritoneal cavity. At week 5 post tapeworm infection, the mice were sacrificed along with uninfected mice, those with only tapeworms, and those with only melanoma cells. A group of uninfected and tapeworm-only infected mice was also sacrificed at week 2 to assess the immune response at the time of the melanoma cell injection.

Tumor growth quantification: In order to quantify the tumor-suppressing effect of the tapeworm infection, the melanoma tumors present in the intraperitoneal cavity, which could be conceivably removed without damaging them or the organs they were attached to, were excised and weighed.

Parasite growth: Five groups of 3 five-week-old C57BL/6J or ICR female mice were also injected intraperitoneally either with *T. crassiceps* or *M. corti* larvae, upon which a group was sacrificed at week 1, 2, 3, 4, or 5 and the larvae were counted.

### 2.5 Histological evaluation

The liver, lungs, and tumors were immediately fixed in 4% neutral-buffered formaldehyde or Bouin’s solution (Sigma-Aldrich). The tissue was then dehydrated in a gradient of ethanol concentrations and embedded in paraffin. Sections of 5 µm were stained with hematoxylin/eosin or Gomori trichrome to visualize small tumors and metastasizing B16F10 cells. For immunohistochemical staining of CD8+ T lymphocytes and NK cells within tumors, the formaldehyde-fixed samples were processed through a saccharose gradient, embedded in a freezing medium (Tissue Freezing Medium, Leica Biosystems), frozen at -80°C and cut into 10 µm sections on a Leica CM 1860 UV cryotome. Afterward, antigen retrieval was performed on the sections, which were boiled in a sodium citrate buffer (10 mM sodium citrate, 0.05% Tween 20, pH 6.0) in a microwave oven at 750 W for 6 minutes. The sections were then incubated overnight at 4°C with either anti-CD8 alpha antibody diluted 1:200 (Abcam, ab237723) or anti-NK1.1 antibody diluted 1:100 (Abcam, ab289542) and visualized by fluorescently-labelled secondary antibodies. The slides were then mounted with 15 µl of Vectashield Antifade Mounting Medium with DAPI (Vector Laboratories), upon which they were examined and photographed using a fluorescent microscope (Olympus BX51 with an Olympus DP-2 camera).

### 2.6 Preparation of antigens

The antigens used consisted of the soluble fraction of whole worm homogenate of *T. crassiceps* (TcH), *M. corti* (McH), or the whole cell homogenate of B16F10 melanoma cell line (MelH). They were prepared by the sonication of tapeworm larvae or cancer cells washed in sterile PBS and suspended in a solution of protease inhibitors (cOmplete ULTRA Tablets, Mini, EDTA-free, EASYpack Protease Inhibitor Cocktail, Sigma-Aldrich) in sterile PBS. After sonication (3x30 second, amplitude 60 W), the homogenate was centrifuged at 16,000 ×g for 20 minutes, upon which the supernatant was collected and passed through a 0.2 µm filter. Protein content was determined, and the homogenates were aliquoted and stored at -80°C.

### 2.7 ELISA

To determine the levels of parasite-specific and B16F10-specific IgM and IgG antibodies in the sera of mice, 96-well plates (Nunc MaxiSorp, Thermo Fisher Scientific) were coated with TcH, McH, or MelH at 2.5 ng/µl in a carbonate-bicarbonate buffer (pH 9.6) overnight at 4°C. After a wash, the wells were blocked with 5% non-fat dried milk in PBS for 1 hour at 37°C. Subsequently, the plates were washed, and sera diluted in the blocking solution (1:500 for IgM and 1:100 for IgG) were added to the wells and incubated for 1 hour at room temperature. Afterward, the plates were rewashed, and goat anti-mouse secondary antibodies conjugated with peroxidase (Anti-Mouse IgM antibody, Abcam ab97230, Anti-Mouse IgG Fc specific antibody, Sigma-Aldrich A2554) diluted in 0.5% bovine serum albumin in PBS (1:10,000 for IgM and 1:8,000 for IgG), were added and incubated for 1 hour at room temperature. Following another wash, peroxidase substrate (TMB – Liquid Substrate System for ELISA, Sigma-Aldrich) was added. The reaction was halted with a stopping solution (2M H_2_SO_4_). The absorbance values were then read at 450 nm using an Infinite M 200 (TECAN) and Magellan software. The cut-off values were determined according to Frey et al. (29) using measurements of sera from mice without any tapeworms. Separate cut-off values were established for 2 week, 5 week and melanoma groups.

### 2.8 Isolation and stimulation of splenocytes

Splenocytes were isolated as described by Majer et al. (30). Briefly, the spleen was mechanically homogenized by passing through a 70μm cell strainer, and the red blood cells were lysed by an ACK buffer. After washing with PBS, the splenocytes were resuspended in RPMI 1640 (supplemented with 10% fetal bovine serum (FBS), 2 mmol/L L-glutamine, 100 U/mL penicillin, and 100 µg/mL streptomycin) and counted (Countess^®^ II Automated Cell Counter, ThermoFisher Scientific). The splenocytes were seeded in 24-well plates at a density of 1.25×10^6^ cells/ml and stimulated by tapeworm (TcH or McH) or melanoma (MelH) antigens (1 and 10 μg/ml). Treatment with concanavalin A (1.25 μg/ml) was used as a positive control for cytokine production. After 72 hours of cultivation (37°C, 5% CO_2_), the supernatants were collected and stored at –20°C until the analysis of cytokine production. Specifically, concentrations of IFN-γ, IL-4, IL-10, and IL-17 were measured by ELISA MAX™ Standard Sets (BioLegend).

### 2.9 Isolation and immunophenotyping of cells in the peritoneal cavity

To isolate the leukocytes, the peritoneal cavity of mice was washed with 10 ml of ice-cold sterile PBS. After centrifugation (5 min, 170 ×g, 4°C), red blood cells were lysed with an ACK buffer, and the remaining cells were washed in PBS and counted (Countess^®^ II Automated Cell Counter, ThermoFisher Scientific). For immunophenotyping, the cells were first incubated with anti-CD16/CD32 antibody (10 min, 4°C) and stained with Zombie Aqua™ Fixable Viability Kit (1:600; 20 min). After washing with 3% FBS/PBS, the cells were incubated (30 min, 4°C) with a mixture of surface marker antibodies as specified in **Table 1**. The samples were measured by LSR II flow cytometer (Becton Dickinson) and analyzed in FlowJo (v. 10.8.1). FMO controls were used as indicated in **Table 1**. The representative gating strategy is shown in **Supplementary Figures 1A, B**.

**Table 1:** Antibodies used for immunophenotyping of the cells in the peritoneal cavity.

Target	Fluorophore	Clone	Vendor	Dilution	FMO
Common					
CD16/CD32	–	93	eBioscience	1:100	–
CD45	APC-eFluor 780	30-F11	eBioscience	1:100	
Lymphoid panel					
CD3	Alexa Fluor 700	17A2	BioLegend	1:100	–
CD4	PerCP-Cy5.5	GK1.5	BioLegend	1:150	–
CD8a	PE-Cy7	53-6.7	BioLegend	1:150	–
CD19	Brilliant Violet 421	6D5	BioLegend	1:100	–
PD-1	PE	29F.1A12	BioLegend	1:80	yes
NK1.1	FITC	PK136	BioLegend	1:80	yes
Myeloid panel					
CD11b	PE-Cy7	M1/70	BioLegend	1:120	yes
CD64	FITC	X54-5/7.1	BioLegend	1:80	yes
Ly6G	APC	1A8	BioLegend	1:100	–
Ly6C	PE-Dazzle 594	HK1.4	BioLegend	1:100	yes
SiglecF	PerCP-Cy5.5	S17007L	BioLegend	1:80	–
PD-L1	PE	10F.9G2	BioLegend	1:80	yes

### 2.10 Collection of excretory-secretory products

In order to obtain excretory-secretory products (ESP), the tapeworm larvae were removed from the peritoneal cavity of a female ICR mouse 3 months after infection, washed 5 times in sterile PBS, and placed in 75 cm^2^ culture flasks (Eppendorf) with DMEM (Dulbecco’s Modified Eagle’s Medium – high glucose, Sigma-Aldrich) supplemented with 100 U/ml of penicillin-streptomycin. The number of larvae per flask was roughly 300 cysticerci for *T. crassiceps* and 2,000 tetrathyridia for *M. corti*. The tapeworm larvae were then cultured at 37°C and 5% CO_2_. Every two days, the medium was aspirated, filtered through a 0.22 μm syringe-mounted filter (Sigma-Aldrich), frozen at -80°C and replaced with a new dose for a total of 14 days, with the medium collected after the first two days being always discarded. The collected medium was then concentrated using a centrifuge filter (Merck-Millipore). For *in vitro* assays with B16F10 cells, the ESPs were separated into fractions according to protein size (<3, 3-10, 10-30, >30 kDa) using centrifuge filters (Merck-Millipore). The concentration of total proteins was determined using a Quant-iT™ Protein Assay Kit (Invitrogen).

### 2.11 Cell culture

The B16F10 mouse melanoma cells were obtained from ATCC (CRL-6475) and routinely cultured in standard conditions (37°C, humidified atmosphere with 5% CO2) in complete DMEM medium (Life Technologies) with 4.5 g/L L-glucose, L-glutamine, and pyruvate, supplemented with 10% fetal bovine serum (Merck) and 0.1% ciprofloxacin (Sigma-Aldrich).

### 2.12 *In vitro* effects of tapeworm ESP on B16F10 melanoma cells

#### 2.12.1 Toxicity

The *in vitro* cytotoxicity of the ESP fractions (<3, 3-10, 10-30, >30 kDa) was assessed by an Alamar Blue assay (Invitrogen) for ESP concentration of 50 µg/ml. Briefly, B16F10 cells were seeded in 96-well plates (2,500 cells per well). The next day, ESP fractions in complete medium (DMEM supplemented with 10% fetal bovine serum (FBS) and antibiotics) were prepared from stock solutions and added to the cells (100 µl per well). A complete medium without ESP was used as a positive control. After 3 days, the medium was aspirated, and the cells were incubated with 50 µl of Alamar Blue solution per well (10% Alamar Blue reagent in DMEM) for 4 hours. The fluorescence (excitation/emission wavelengths of 560/590 nm) was measured using an Infinite M200 PRO fluorescent plate reader (TECAN). The average results of four independent experiments of the treated cells were plotted relative to the positive control (100% cell viability).

#### 2.12.2 Proliferation

The kinetic measurement of cell growth was performed using holographic microscopy. B16F10 cells were seeded in 96-well plates (Lumox multiwell, Sarstedt; 5,000 cells per well). The next day, ESP fractions (final concentration 50 µg/ml) in complete medium (DMEM supplemented with 10% FBS and antibiotics) were prepared from stock solutions and added to the cells (170 µL per well). A complete medium without ESP was used as a positive control, and two concentrations of Vincristine (VCR) were used as negative controls. The wells were covered with HoloLid (PHI AB). Cell count measurement was performed on a HoloMonitor microscope (PHI AB) using App Suite Imaging Software (PHI AB). Each time point in each condition represents the mean of cell counts of four positions. Two independent experiments were performed.

#### 2.12.3 Wound healing

A suspension of 1×10^5^ B16F10 cells was added to each well of a 24-well plate. The next day, a scratch was made using a pipette tip. The cells were washed two times, upon which ESP fractions in complete medium (DMEM supplemented with 10% FBS and antibiotics) were added (final ESP concentration 50 µg/mL). A complete medium without ESP was used as a positive control. After two days, images of four different fields were acquired for every condition using phase contrast microscopy on a Leica DMi8 microscope under 10× magnification. The percentage of the cell-free area was calculated using the Wound_healing_size_tool plugin (31) for ImageJ. The healed area was subsequently calculated and is shown as the mean of four fields for two biological replicates.

#### 2.12.4 Gelatin degradation assay

A gelatin degradation assay was performed according to manufacturer instructions (QCM Gelatin Invadopodia Assay, Merck-Millipore). B16F10 cells were resuspended in complete medium (DMEM supplemented with 10% FBS and antibiotics) with diluted ESP fractions (final concentration 50 µg/mL), seeded on gelatin for 16 hours, fixed, and stained for actin with Alexa Fluor 488 Phalloidine (ThermoFisher Scientific) and for nuclei with DAPI. Fluorescent images were acquired on a Leica DMi8 equipped with a Leica 100×/1.44 NA oil objective. Gelatin degradation was quantified by measuring the area of degradation and scoring it as a percentage of the total cell area. At least 40-50 cells per condition were scored.

### 2.13 Statistical analysis

The statistical analysis of cytotoxicity, proliferation, gelatin degradation and wound healing of ESPs-affected B16F10 cells was performed by ordinary one-way ANOVA with Dunnett’s multiple comparisons test. The growth of peritoneal tumors, titration of melanoma antigen and the production of cytokines by restimulated splenocytes was analyzed by the Kruskal-Willis test with Dunn’s multiple comparisons test. The growth curves of tapeworms in the peritoneal cavity were analyzed by a two-way ANOVA. Cytometric data were analyzed by one-way ANOVA with Šíďák *post-hoc* test. Statistical analysis was performed in GraphPad Prism 9. Significance level p <0.05.

## 3 Results

### 3.1 Melanoma suppression in mouse models

**Figure 1:**
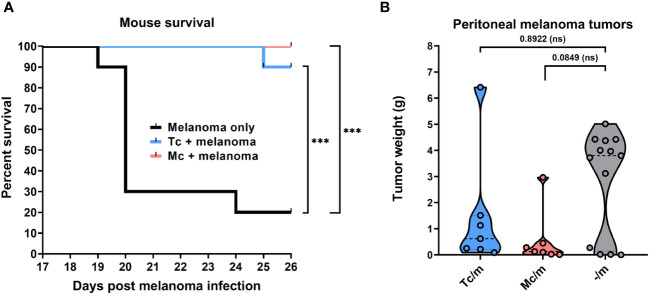
The suppressive effect of tapeworm infection on melanoma. The overall survival of C57BL/6J mice infected with *T. crassiceps* and *M. corti* (each at n = 10) shows significant improvement in both cases of tapeworm infection compared to mice injected with only melanoma when analyzed with the Log-rank test **(A)**. The weight of peritoneal tumors reveals a trend of suppression on their growth (n = 7), showing a greater decrease of peritoneal tumors in *M. corti* infections of C57BL/6J mice after analysis with Kruskal-Willis test with Dunn’s multiple comparisons **(B)**. *** (p ≤ 0.001), ns (no significance).

Both the *Taenia crassiceps* and *Mesocestoides corti* infections significantly improved the survival of C57BL/6J mice with intraperitoneally administered B16F10 melanoma cells compared to those injected with melanoma only. While only 2 out of 10 melanoma-only mice survived after 26 days, all the mice also infected with *M. corti*, and 9 out of 10 infected with *T. crassiceps* survived until the end of the observation period (**Figure 1A**).

**Figure 2:**
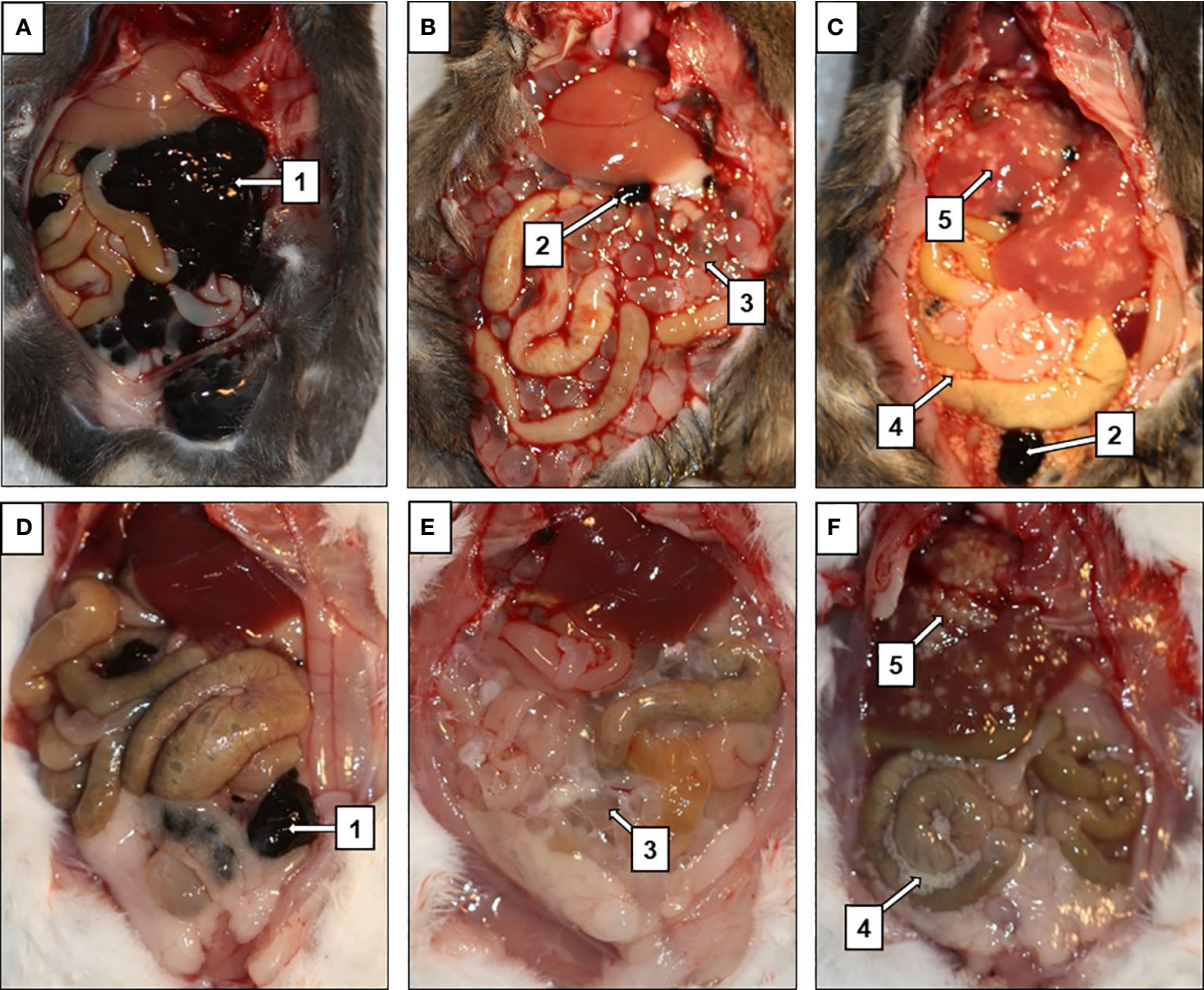
Suppression of melanoma tumor growth in mice infected with tapeworms. In mice injected with only melanoma **(A, D)**, the entirety of the peritoneal cavity was filled with masses of tumors (1), while only few smaller tumors (2) were found in mice infected with *T. crassiceps* **(B, E)** or *M. corti* **(C, F)**. *T. crassiceps* cysticerci (3), free *M. corti* tetrathyridia (4), *M. corti* tetrathyridia embedded in liver parenchyma (5). **(A–C)** are C57BL/6J mice, **(D–F)** are ICR mice.

The tumor-suppressive effect (**Figure 2**) was also quantified by weighing the melanoma tumors found within the peritoneal cavity of infected mice and comparing these weights with those from melanoma-only mice. The results show only a trend in the reduction of melanoma growth in the peritoneal cavity, as due to high variability of the data, the results were not significant. The burden of tumors tended to be lower in C57BL/6J mice infected with *M. corti* larvae when compared with the *T. crassiceps* infection (**Figure 1B**). The results were not as pronounced in ICR mice, as the intraperitoneal melanoma burden was very low even in mice without any tapeworm larvae (**Supplementary Figure 2**, **Supplementary Table 2**).

In the case of the C57BL/6J strain (**Figures 2A–C**), mice infected with *M. corti* developed only small tumors in the peritoneal cavity and liver (**Figure 2C**), but none in the lungs. In particular histological examination of the liver revealed some melanoma cells. Still, they were mostly localized at the margin and not spread throughout the entire tissue, as in mice bearing only melanoma (**Supplementary Figure 3**). In the case of *T. crassiceps*, the number of melanoma tumors in the peritoneal cavity was greater compared to *M. corti* infections (**Figure 1B**). The tumors were found in the liver and lungs, indicating a weaker anti-tumor effect elicited by *T. crassiceps* (**Supplementary Figure 3**).

**Figure 3:**
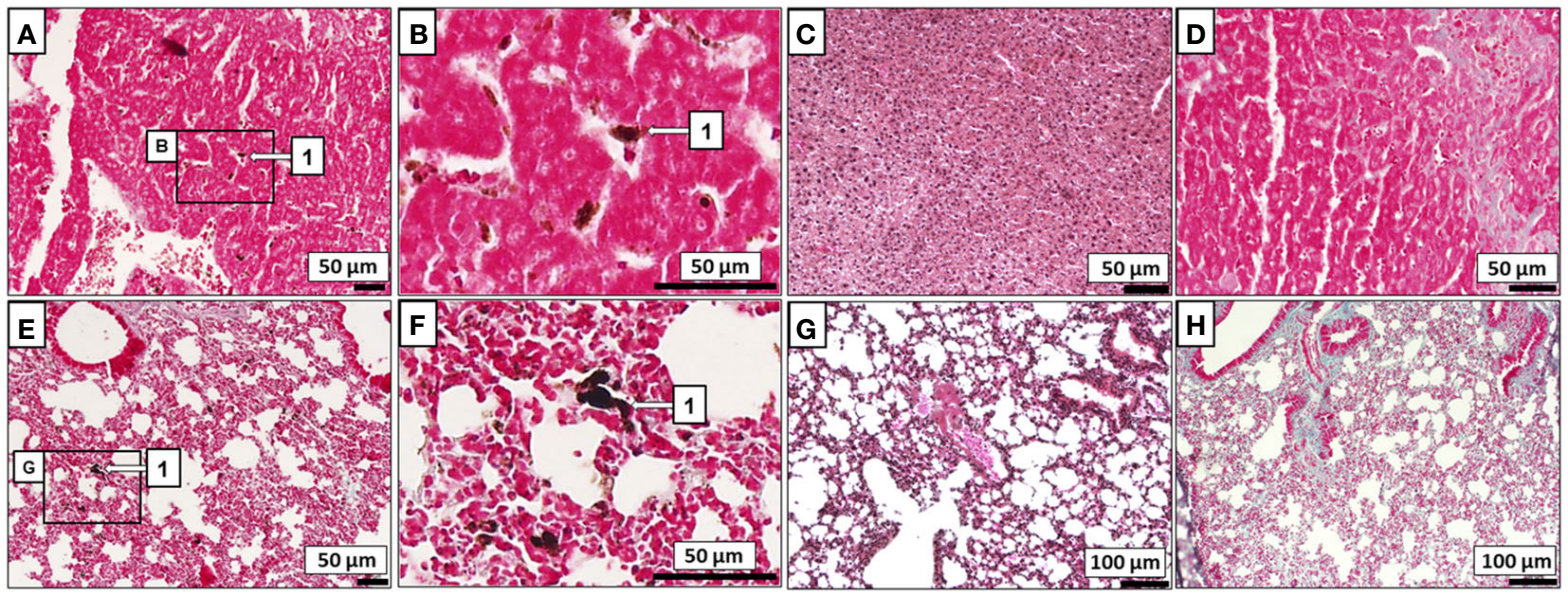
Histological evaluation of melanoma metastasis. B16F10 cells (1) were found to invade the liver **(A)** and lungs **(E)** of ICR mice without any tapeworm infection, while none were found within the liver **(C, D)** or lungs **(G, H)** of ICR mice infected also with *T. crassiceps M. corti*. Zoomed-in areas of the liver **(B)** and the lungs **(F)** with melanoma.

Most ICR mice did not develop macroscopic melanoma tumors (**Figures 2E, F**) or exhibit lung or liver metastatic cells when infected with *T. crassiceps* (**Figures 3C, G**) or *M. corti* (**Figures 3D, H**). In mice injected with melanoma only, the entire peritoneal cavity was usually filled with foci of melanoma tumors surrounding the liver (**Figure 2D**), and macroscopic tumors formed in the lungs as well. Histological sections revealed metastatic melanoma cells in the liver and lungs of tapeworm-uninfected mice (**Figures 3A, B, E, F**).

### 3.2 Tapeworm growth rate

**Figure 4:**
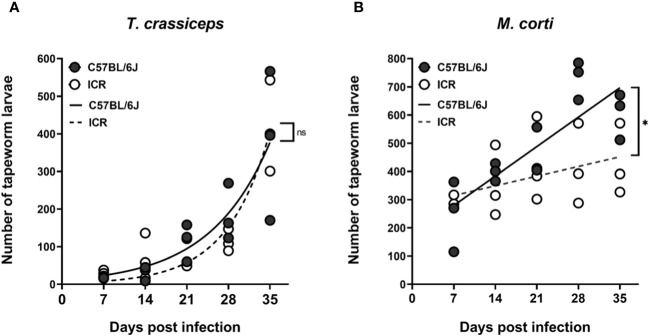
The growth of tapeworm larvae in the peritoneal cavity. Graph **(A)** shows that there is no difference in the growth curves of *T. crassiceps* between the C57BL/6J and ICR mouse strains, while the growth of *M. corti* **(B)** is slower in ICR mice compared to C57BL/6J mice. Each timepoint group consisted of n = 3, at a total of 5 groups per infection and mouse strain. A two-way ANOVA was utilized to compare the growth curves. ns (no significance), * (p ≤ 0.05).

Tapeworm larvae present intraperitoneally were counted throughout five weeks in one-week intervals, and the growth rates of particular tapeworm species in both mouse strains were compared. *T. crassiceps* cysticerci grew exponentially (**Figure 4A**), while *M. corti* tetrathyridia grew more linearly (**Figure 4B**). When comparing the growth data with 2-way ANOVA, there was no difference in the growth rates of *T. crassiceps* in C57BL/6J and ICR mice, while the growth rate of *M. corti* was slower in ICR mice. It does not appear that an increased tapeworm burden would be responsible for the slower growth of melanoma in ICR mice compared to C57BL/6J.

### 3.3 Antibody response to tapeworm antigens and possible cross-reactivity

**Figure 5:**
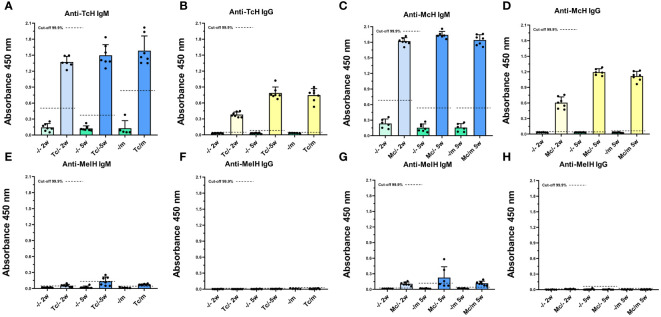
Antibody response to tapeworm and melanoma antigens. C57BL/6J mice infected with *T. crassiceps* **(A, B, E, F)** or *M. corti* **(C, D, G, H)** (n = 7), are given here as an example. The sera of mice infected with tapeworms contained high levels of specific IgM **(A, C)** and IgG **(B, D)** raised against the antigens of the respective tapeworm, while they did not contain any significant amount of IgM **(E, G)** or IgG **(F, H)** specific to MelH. The cut-off values were determined according to Frey et al. (29).

The levels of IgM and IgG antibodies, which bind to either TcH, McH, or MelH, were measured in the sera of all mice. The sera of tapeworm-infected mice of both mouse strains showed high levels of specific IgM (**Figures 5A, C**) and IgG (**Figures 5B, D**) raised against the antigens of the respective tapeworms. On the contrary, in melanoma-positive mice, the specific anti-MelH IgG was not detected in any of the sera (**Figures 5F, H**). Only low IgM levels that reacted to MelH antigen were detected, mainly in tapeworm-infected C57BL/6 sera (**Figures 5E, G**). An antigen titration was performed to investigate these antibodies further, where the MelH concentration was increased up to 20x of the base level of 2.5 ng/µl. The titration did not significantly increase detected MelH-specific IgM at 5x or 10x, but only at the 20x mark, when compared with the base level, showing that the binding is likely non-specific (**Supplementary Figure 4**). Antibody levels for ICR mice are shown in **Supplementary Figure 5**.

### 3.4 Host immune response in the peritoneal cavity

**Figure 6:**
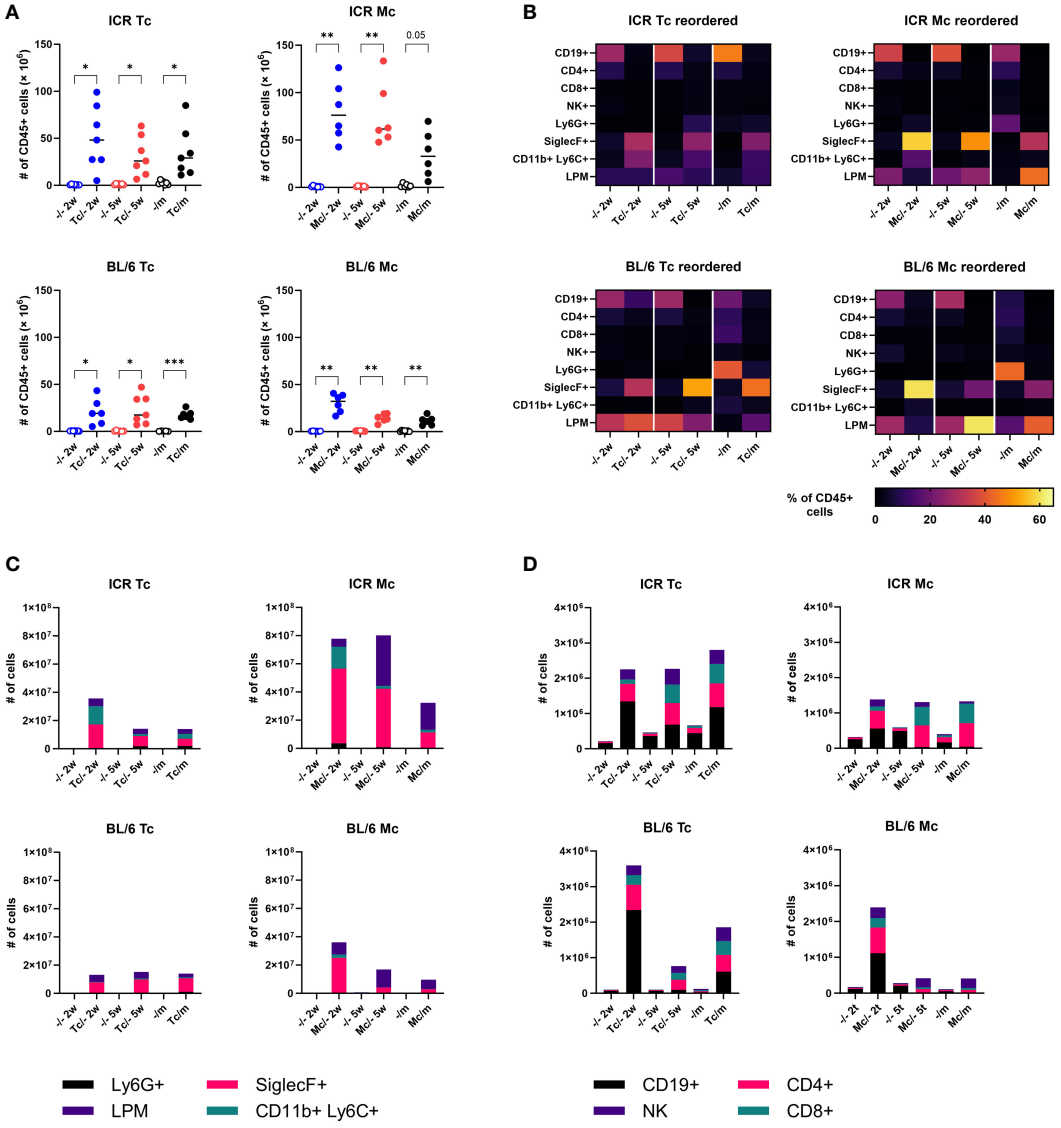
Host immune response in the peritoneal cavity of mice. **(A)** Total numbers of leukocytes. Dots represent data from individual mice (n = 7); group medians are shown. **(B)** Heat maps showing the frequency of major leukocyte populations. **(C, D)** Total cell counts of major myeloid **(C)** and lymphoid **(D)** populations. Medians are shown; error bars are omitted to keep legibility. CD19+, B cells; CD4+, T helper cells; CD8+, cytotoxic T cells; NK+, natural killer cells; Ly6G+, neutrophils; SiglecF+, eosinophils; CD11b+Ly6C+, monocytes; LPM, large peritoneal macrophages. One-way ANOVA with Šíďák *post-hoc* test was used to analyze the data. * (p ≤ 0.05), ** (p ≤ 0.01), *** (p ≤ 0.001).

To better understand the cellular host immune response, which could affect the course of the infection and melanoma growth and spreading, we performed flow cytometry analysis of cells in the peritoneal cavity. We revealed a massive accumulation of leukocytes in the peritoneal cavity of infected mice, especially 2 weeks post infection (w.p.i.) (**Figure 6A**). Interestingly, infection with *M. corti* triggered a more pronounced cellular inflammation, and infected ICR mice had 2-fold more leukocytes in the peritoneal cavity. While B lymphocytes were most frequent in the peritoneal cavity of healthy mice, neutrophils prevailed in mice with melanoma, and eosinophils predominated in tapeworm-infected mice, either with or without melanoma (**Figures 6B, C**). The detailed analysis of lymphoid cell counts (**Figure 6D**) revealed that the usual anti-tumor suspects, NK cells and CD8+ cytotoxic T lymphocytes, were present in the peritoneal cavity at the time of melanoma inoculation (at 2 weeks) as well as when the experiment was terminated (at 5 weeks). Therefore, we sought to histologically locate both NK and CD8+ cytotoxic T lymphocytes within melanoma tumor tissue. However, none were found in either tumor from mice without a tapeworm infection or those with tapeworms (data not shown). Beyond the leukocyte quantities, we also monitored their functional markers and response in the peritoneal cavity and on the systemic level. As for the expression of immunoregulatory checkpoints PD-1 and PD-L1, the tapeworm infection increased the proportion of PD-1+ cytotoxic T lymphocytes and PD-L1+ eosinophils (data not shown), suggesting active manipulation of the host immune response by the parasites. On the systemic level, we detected tapeworm-specific production of IL-4, IL-10, and IFN-γ by splenocytes isolated from infected mice, but no melanoma-specific cytokine response was observed (**Supplementary Figure 6**).

### 3.5 Cytotoxicity of ESPs

**Figure 7:**
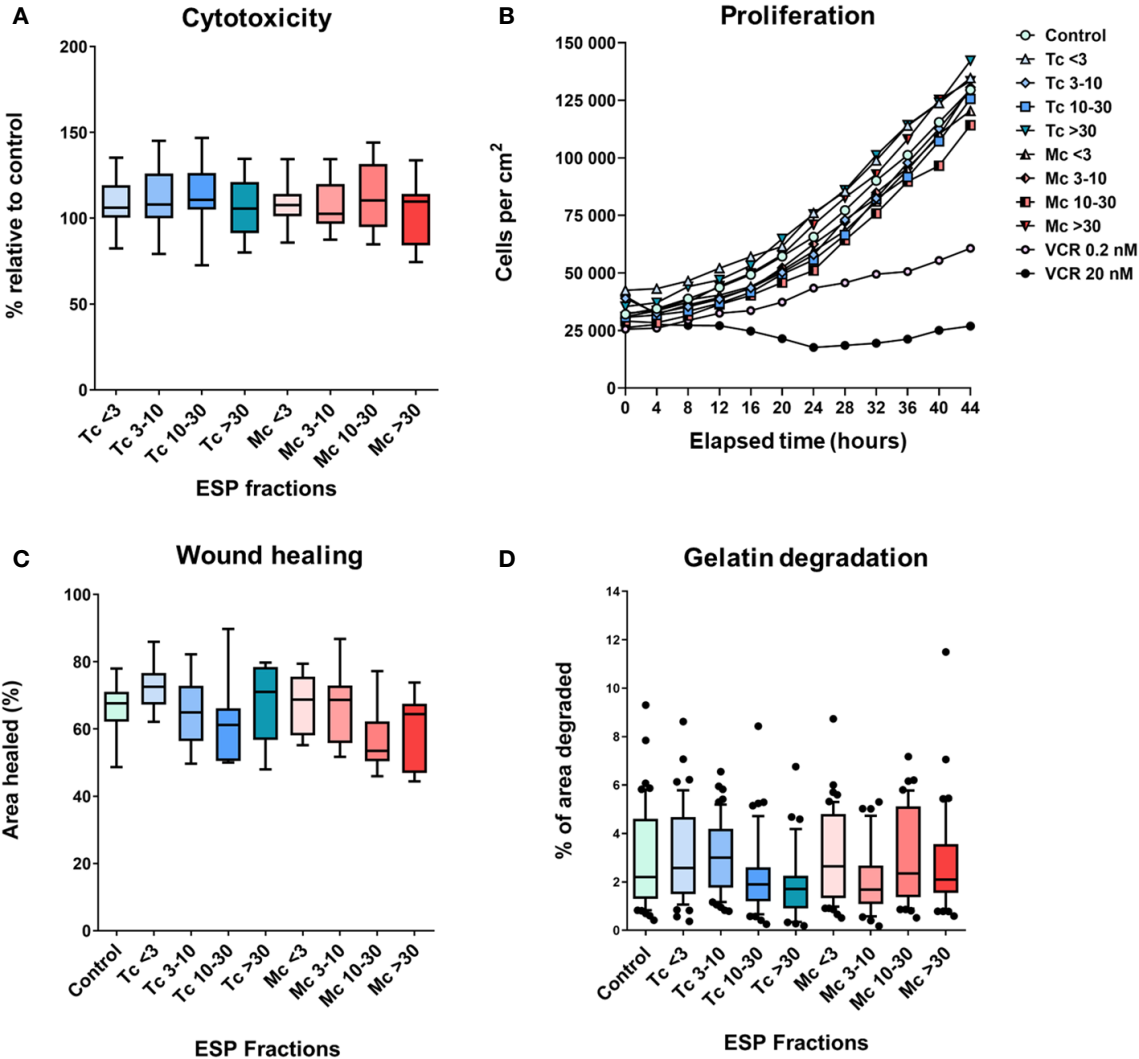
The *in vitro* effect of tapeworm excretory-secretory products on B16F10 cells. Graphs **(A, B)** show the cytotoxic effect, where **(A)** shows the overall survival of cells compared to control, while **(B)** shows the effect on the cells’ ability to grow. Graphs **(C, D)** focus on their invasive attributes. The ESPs’ effect on their migration capabilities **(C)** and on their ability to degrade the extracellular matrix (gelatin) **(D)** (<3, 3-10, 10-30 and >30 denote the fractions of ESPs in kDa, VCR = vincristine). The results were analyzed with a one-way ANOVA with Dunnett’s multiple comparisons test.

To explore the possible direct effect of ESPs on cancer cells, we analyzed their effect on B16F10 melanoma cell proliferation, migration in 2D, and the ability of B16F10 cells to degrade extracellular matrix. The cytotoxicity of ESPs was tested by an endpoint assay (Alamar Blue assay) and a holographic microscopy-based kinetic assay. In both settings, ESPs did not affect cell growth (**Figures 7A, B**). The effect of ESPs on cell migration was analyzed using scratch wound assay. None of the ESP fractions significantly changed the ability of B16F10 cells to heal the wound (**Figure 7C**). Finally, since B16F10 cells use a mesenchymal type of invasion dependent on the degradation of the extracellular matrix (32, 33), we analyzed the effect of ESPs on the ability of B16F10 cells to degrade gelatin. As in previous analyses, no effect of ESPs was observed (**Figure 7D**). Altogether, these *in vitro* experiments indicate that ESPs are unable to directly affect the growth and pro-invasive characteristics of B16F10 cancer cells.

## 4 Discussion

Several studies indicate an ambivalent effect of helminth infections on cancer development. In our case, *T. crassiceps* and *M. corti* larvae induced slower B16F10 melanoma tumor growth in mice. Here we aim to explain the interesting effect of tapeworm larvae infection on melanoma tumors in mice.

The experiments were designed to allow us to explore both the melanoma growth suppression and the possible explanations for the effect. First, the number of inoculated B16F10 cells was lowered compared to the survival experiments, to ensure mouse well-being until the point of examination, three weeks post-injection. Second, we have introduced the B16F10 cells intraperitoneally, as the suppressive effect is locked to that site – in our preliminary experiments, the infections did not remotely suppress melanoma introduced intravenously or subcutaneously (data not shown). Third, the B16F10 cells were inoculated 2 weeks after the tapeworms, to allow the larvae sufficient time to establish an infection. Based on this, we have shown the suppressive effect through a significantly improved survival rate in C57BL/6J mice with both tapeworms and melanoma, compared to mice with only melanoma, and by showing a reduction in melanoma tumor weight in mouse peritoneal cavities. We only performed the former with C57BL/6J mice, as a similar dose of B16F10 cancer cells is not lethal to the ICR strain.

Considering the slower tumor growth in ICR mice compared to C57BL/6, we primarily presumed that a greater amount of tapeworm larvae in the ICR mice peritoneal cavity might be responsible for tumor growth suppression, due to a higher amount of released ESPs and/or a stronger immune response. Thus, the growth curves of both tapeworms were measured. However, they have shown no difference in *T. crassiceps* growth between the mouse strains, and the growth of *M. corti* is actually slower in ICR mice. Since ICR mice do not bear a higher number of tapeworm larvae than C57BL/6J mice, the slower tumor growth cannot correlate with parasite burden; the difference in tumor growth is, therefore, probably attributable to other factors, such as the variability between mouse strains. The B16F10 melanoma cell line was originally isolated from the C57BL/6 mouse strain (24) and, therefore, is syngeneic for this strain while being allogeneic for the ICR mice. Previous research has also shown that the B16F10 melanoma tumors grow slower in the ICR strain than C57BL/6J (34).

While previous research proved the existence of antibodies that cross-react with antigens from both helminth parasites and tumor cells (35), we have not detected any that would cross-react with antigens from the B16F10 melanoma cell line, and either of the tapeworms. Although both *T. crassiceps* and *M. corti* do express the Tk antigen, which is also found in certain types of cancer cells (36), the absence of cross-reactivity in our experiments indicates that the shared antigens are either not expressed in the B16F10 melanoma cells or no specific antibodies are produced in the respective mouse strains. Furthermore, B16F10 cells are known to have low immunogenicity due to a decreased MHC I expression (37). A certain level of IgM binding to MelH was detected; however, the antibody binding did not significantly rise when MelH concentration was increased. We have performed this experiment with C57BL/6J sera only, since per our Western blot experiments (data not shown) antibodies from both mouse strains recognize the same *T. crassiceps* or *M. corti* antigens after a tapeworm infection. Furthermore, levels of antibodies are more similar in this inbred strain compared to the outbred ICR (**Figure 5**, **Supplementary Figure 5**).The reaction was most likely caused by the greater propensity of IgM to bind non-specifically to the components of MelH due to its lower specificity when compared with IgG (38). We can summarize, therefore, that the presence of antibodies raised against tapeworm antigens is likely not responsible for the suppressive effect these infections have on the subsequently introduced melanoma cells.

Due to the different levels of tumor suppression in ICR and C57BL/6J, we suspected that distinct immunological backgrounds of the mice could play a role. Splenocytes from mice treated with certain peptides isolated from *E. granulosus* have a cytotoxic effect on pancreatic cancer cells (39). In our experiments, restimulation of splenocytes with B16F10 melanoma antigen did not show any effect in either mouse strain. Therefore, the tumor-suppressing effect is probably not mediated by splenocytes or T cells reacting to potentially shared tapeworm-melanoma antigens. Flow cytometry analysis of peritoneal cavity immune cells revealed a significant increase in leukocytes associated with anti-tumor immunity in infected mice. For example, NK cells are an important part of cancer immunotherapy (40) and were shown to promote protection against melanoma liver metastasis in C57BL/6J mice (41). They are proposed as a possible effector in tapeworm-mediated tumor suppression in *E. granulosus* infections (42). The numbers of NK cells were elevated in both *T. crassiceps*- and *M. corti*-infected mice, mainly in the ICR strain. The CD8+ T cell population was also increased in infected mice and could contribute to tumor growth suppression. These population patterns of particular immune cells do not entirely align with the observed levels of melanoma suppression in different mouse strains. Moreover, the immunohistochemistry of tumors isolated from the peritoneal cavity of infected mice also did not show any NK or CD8+ T cell infiltrates, nonetheless, it is possible that the effect is only capable of destroying solitary cells. The amount of injected cells might overwhelm the immune system, allowing some to escape, develop immune evasion and form tumors, which are more difficult to destroy due to the presence of immunosuppressive cells (43). It could also mean, however, that other cells are responsible for the effect. For example, eosinophils were responsible for tumor growth reduction in *Hymenolepis nana*-infected mice (44). These cells were the dominant cell population in our experiments, thus, hypothetically, the toxic products excreted by eosinophils and targeted at the tapeworm larvae could also damage the melanoma cells. Eosinophils could also produce extracellular traps primarily used against pathogens (45) that might be used against migrating tumor cells.

Besides the direct effect of the immune cells on tumors, we also admit other immunity-unrelated mechanisms. For example, the strong inflammation at the beginning of the tapeworm infection in the peritoneum could lead to a depletion of nutrients, which could affect the ability of the melanoma cells to survive (46), besides the nutrient consumption by the tapeworms themselves. As tapeworms are known to produce some organic acids due to anaerobic glucose metabolism, e.g., lactate, (47), we indicatively measured the intraperitoneal pH of tapeworm-infected and uninfected mice to ascertain whether the environment into which the cancer cells are introduced is hostile for reasons other than the immune response. The pH, however, was the same in the uninfected mice and both infections, hovering around 7.4 (data not shown).

To evaluate the direct effect of excretory-secretory products on cancer cells, we used B16F10 cells treated with ESPs from both *T. crassiceps* and *M. corti*. We used four fractions separated according to molecular weight (<3 kDa, 3-10 kDa, 10-30 kDa, and >30 kDa). First, we tested whether the ESPs have any direct cytotoxic effect on cancer cells by an endpoint assay and kinetic assay. Neither of these methods made a significant difference in cell growth.

The formation of metastases is influenced by the growth of tumor cells and their ability to spread in the body. Since we did not detect any cytotoxicity of ESPs, we wanted to test whether ESPs affect the metastatic potential of tumor cells. Cancer cell invasion is a crucial step in metastasis formation (48, 49), a multistage process composed of phenotypic and biochemical changes. During the first step of metastatic spreading, the malignant tumor cells initiate separation from the primary tumor mass and break contact with neighboring cells. Then, the tumor cells degrade and penetrate the extracellular matrix and enter the bloodstream or lymphatic system, from which they can exit at a new site and proliferate in other organs (50, 51). The scratch wound healing assay is a widely used method for testing the effects of various compounds on cellular migration. Our wound healing tests found no significant change in the migration of cells treated with ESPs compared to control cells. Since tumor cells’ invasive and metastatic potential depends not only on migration but also on their capability to degrade the extracellular matrix, we employed a gelatin degradation assay. Again, no effect of ESPs on gelatin degradation was observed. Thus, we conclude that the ESPs, and probably the infection by tapeworms, most likely do not affect the cancer cells directly.

To sum up, our paper introduces larvae of *M. corti* as a new model to study the effect of tapeworm infection on cancer development in mice. A B16F10 tumor-suppressing effect of *M. corti* and *T. crassiceps* infection upon intraperitoneal application of melanoma cells was demonstrated with a different degree of effectiveness in different strains of mice. Our findings suggest the involvement of the local peritoneal immune system activated by tapeworm infection. However, further studies are required to characterize the local processes (both immunological and immunity-unrelated) facilitating the protective effect of tapeworm infection against melanoma. Overall, the effect seems more preventative, rather than capable of reducing established melanoma. Nonetheless, as we plan to isolate and test particular tapeworm molecules produced and administer them in higher concentrations intraperitoneally or near tumors, we could also reduce their growth.

## Data availability statement

The raw data supporting the conclusions of this article will be made available by the authors, without undue reservation.

## Ethics statement

The animal study was approved by Animal Welfare Committee of Charles University, Faculty of Science. The study was conducted in accordance with the local legislation and institutional requirements.

## Author contributions

MS: Conceptualization, Formal analysis, Investigation, Methodology, Writing – original draft, Writing – review & editing. TM: Conceptualization, Formal analysis, Investigation, Methodology, Visualization, Writing – original draft, Writing – review & editing. VV: Formal analysis, Investigation, Methodology, Visualization, Writing – original draft, Writing – review & editing. BŠ: Formal analysis, Investigation, Writing – review & editing. MM: Investigation, Methodology, Writing – review & editing. JH: Investigation, Visualization, Writing – review & editing. OT: Conceptualization, Formal analysis, Investigation, Visualization, Writing – original draft, Writing – review & editing. JB: Supervision, Writing – review & editing. DR: Supervision, Writing – review & editing. PH: Conceptualization, Funding acquisition, Writing – review & editing.

## Supplementary material

Supplementary Figure 1 Representative gating strategy in the peritoneum with fluorescence minus one (FMO) controls.

Supplementary Figure 2 Melanoma tumor weight in the peritoneum of ICR mice. The growth and weight of melanoma was very low even in non-infected ICR mice (n = 7) and, therefore, cannot provide suitable results for statistical analysis.

Supplementary Figure 3 Histological evaluation of melanoma metastasis in C57BL/6J mice. B16F10 cells (1) were found to invade the lungs **(A)** and the liver **(B)** of C57BL/6J mice without any tapeworm infection. In *T. crassiceps* infections, melanoma were also found in the lungs **(D)** and the liver **(E)**, while in *M. corti* infections, they were only found in the liver **(H)**, while the lungs were clear of them **(G)**. Zoomed-in areas of the liver metastases **(C, F, I)**.

Supplementary Figure 4 Titration of MelH antigen. There was significant change in IgM binding to MelH only when the antigen concentration was increased 20x, showing that the IgM probably bound to the antigen only in a non-specific manner, after analysis with Kruskal-Willis test with Dunn’s multiple comparisons. C57BL/6J mouse sera were tested (n = 5), infected with either *T. crassiceps* or *M. corti*.

Supplementary Figure 5 Antibody response to tapeworm and melanoma antigens of ICR mice infected with *T. crassiceps* and M*. corti*. Here, the mouse strains and infections are as follows: **A – D** ICR mice infected with *T. crassiceps*, **E – F** ICR mice infected with M. corti. Sera of all infected mice contained high levels of specific IgM **(A, E)** and IgG **(B, F)** raised against the antigens of the respective tapeworm. In contrast, they did not contain any significant amount of IgM **(C, G)** or IgG **(D, H)** specific to MelH. (n = 7) The cut-off values were determined according to Frey et al. (29).

Supplementary Figure 6 Cytokines produced by restimulated splenocytes. An example of cytokine production by splenocytes restimulated with tapeworm homogenate or melanoma homogenate, ICR mice infected with *T. crassiceps* (n = 7) given as example. Values below detection limit and those above the calibration curve are omitted. – represents control, + concanavalin, MelH 1 and MelH 10 represent 1 and 10 μg/ml of melanoma cell homogenate, TcH 1 and TcH 10 then 1 and 10 μg/ml of *T. crassiceps* homogenate. Statistical analysis was performed with Kruskal-Willis test with Dunn’s multiple comparisons test.
