# Supplementary material for: Suppression of the growth and metastasis of mouse melanoma by Taenia crassiceps and Mesocestoides corti tapeworms
Source: Front Immunol. 2024 Mar 20;15:1376907. doi: 10.3389/fimmu.2024.1376907 (PMC10987685; doi:10.3389/fimmu.2024.1376907)
Supplement: Supplementary Figure 1 — Representative gating strategy in the peritoneum with fluorescence minus one (FMO) controls. [file Image_1.pdf]

LYMPHOID CELLS

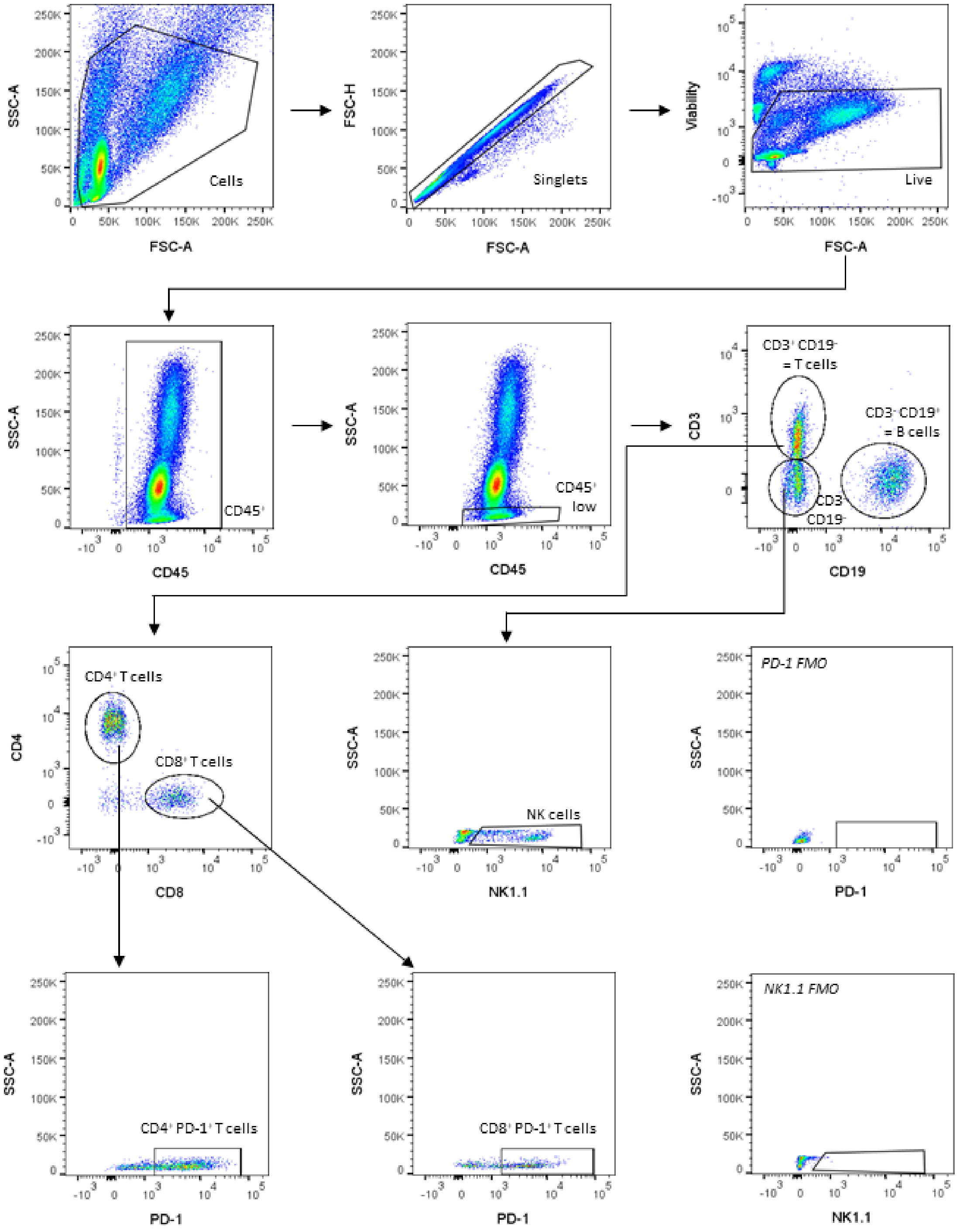

MYELOID CELLS

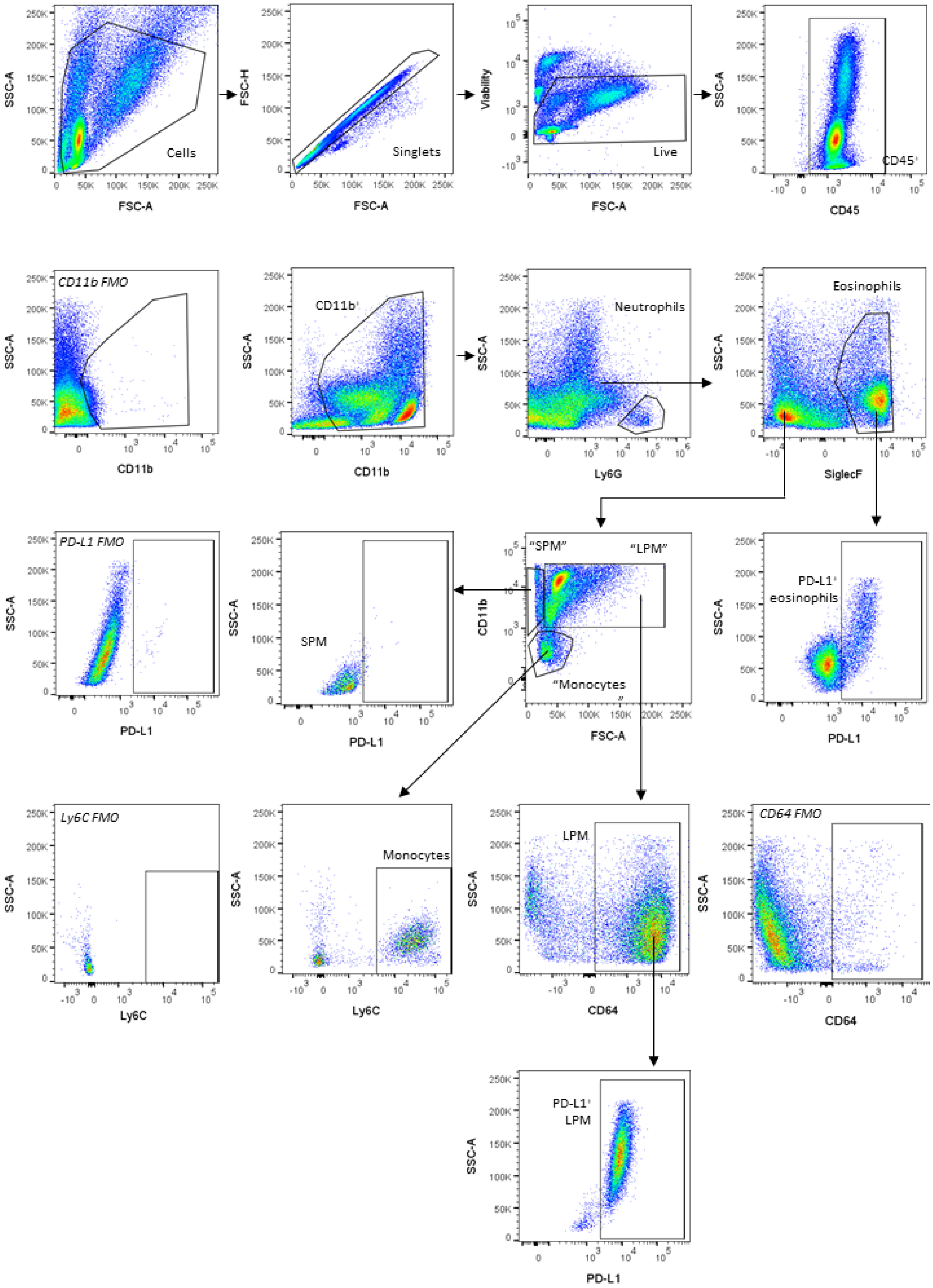

SFig. 1. Representative gating strategy in the peritoneum with fluorescence minus one (FMO) controls.
