# Supplementary material for: Suppression of the growth and metastasis of mouse melanoma by Taenia crassiceps and Mesocestoides corti tapeworms
Source: Front Immunol. 2024 Mar 20;15:1376907. doi: 10.3389/fimmu.2024.1376907 (PMC10987685; doi:10.3389/fimmu.2024.1376907)
Supplement: Supplementary Figure 2 — Melanoma tumor weight in the peritoneum of ICR mice. The growth and weight of melanoma was very low even in non-infected ICR mice (n = 7) and, therefore, cannot provide suitable results for statistical analysis. [file Image_2.pdf]

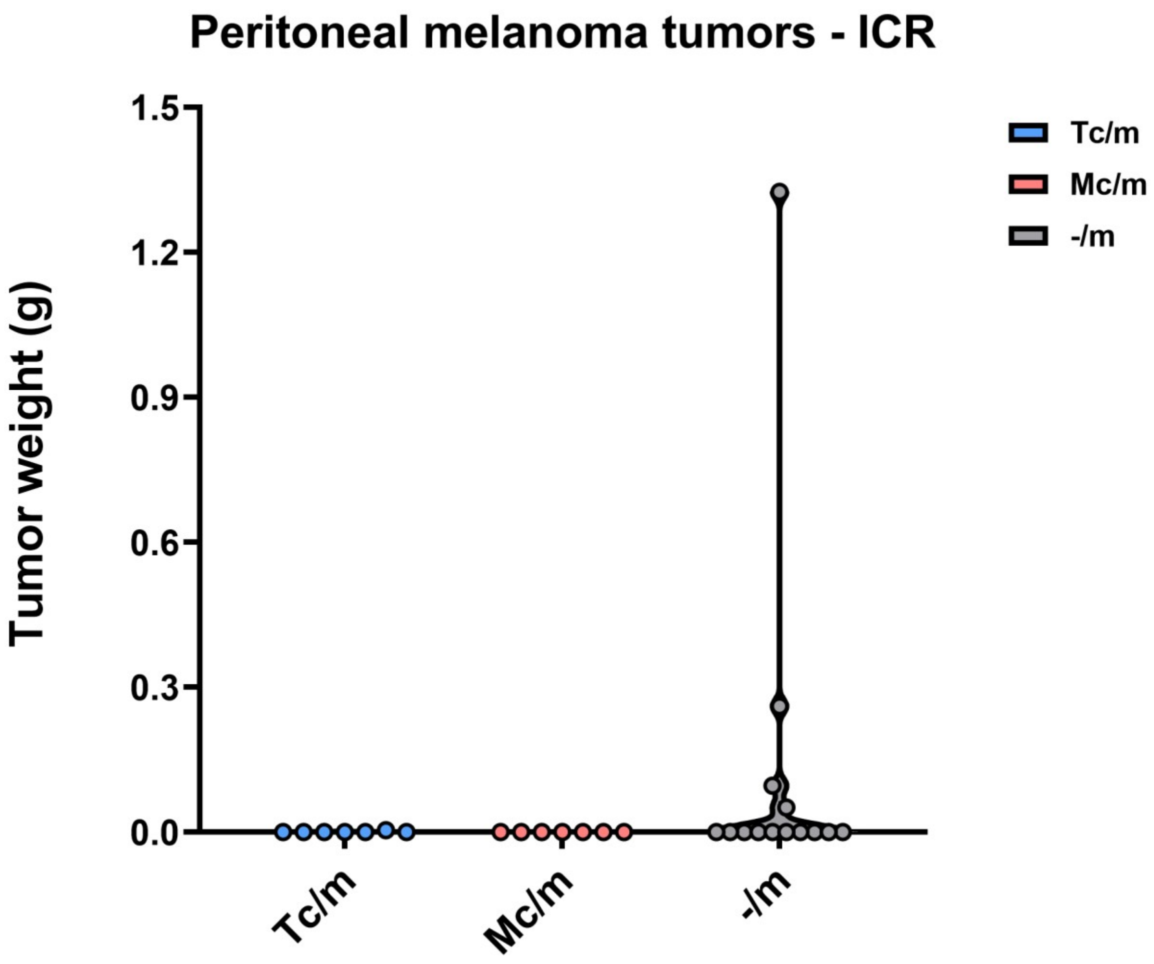

SFig. 2. Melanoma tumor weight in the peritoneum of ICR mice. The growth and weight of melanoma was very low even in non-infected ICR mice (n = 7) and, therefore, cannot provide suitable results for statistical analysis.
