# Supplementary material for: Suppression of the growth and metastasis of mouse melanoma by Taenia crassiceps and Mesocestoides corti tapeworms
Source: Front Immunol. 2024 Mar 20;15:1376907. doi: 10.3389/fimmu.2024.1376907 (PMC10987685; doi:10.3389/fimmu.2024.1376907)
Supplement: Supplementary Figure 3 — Histological evaluation of melanoma metastasis in C57BL/6J mice. B16F10 cells (1) were found to invade the lungs (A) and the liver (B) of C57BL/6J mice without any tapeworm infection. In T. crassiceps infections, melanoma were also found in the lungs (D) and the liver (E), while in M. corti infections, they were only found in the liver (H), while the lungs were clear of them (G). Zoomed-in areas of the liver metastases (C, F, I). [file Image_3.pdf]

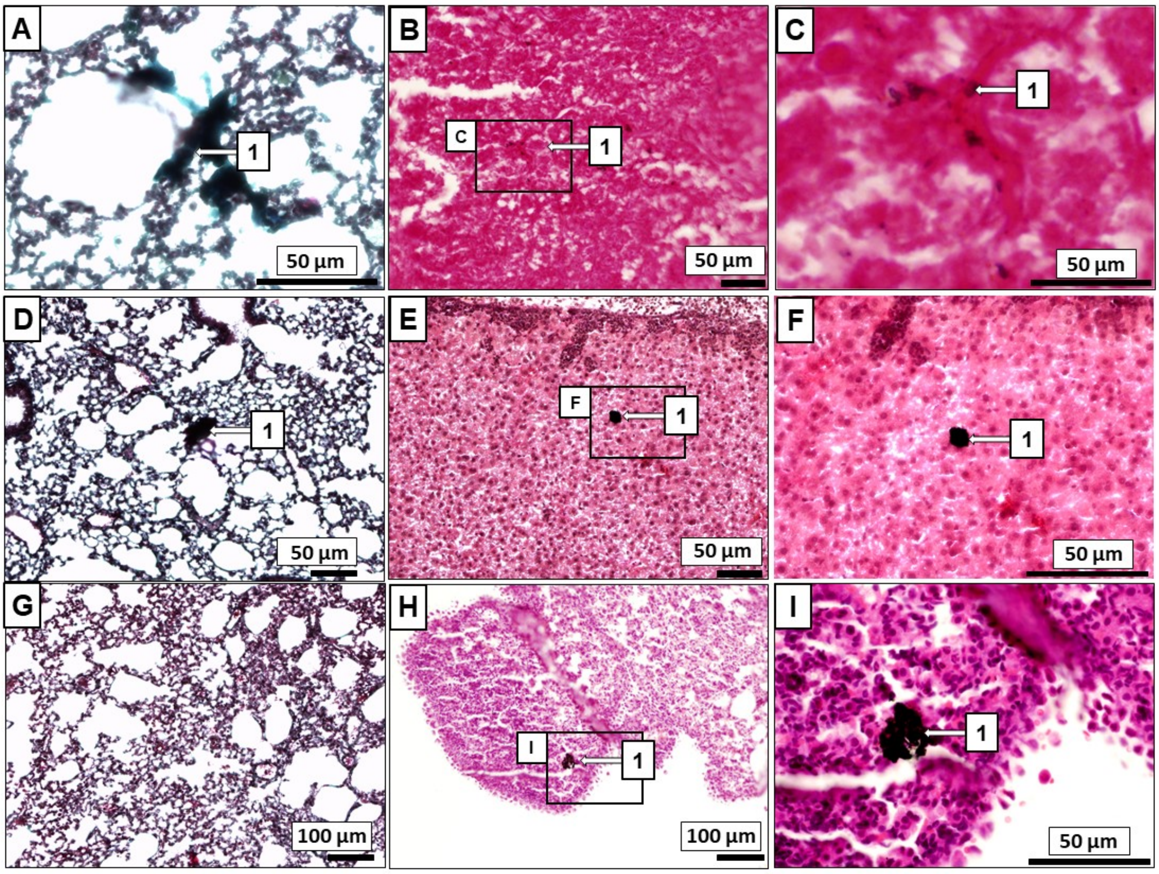

SFig. 3. Histological evaluation of melanoma metastasis in C57BL/6J mice. B16F10 cells (1) were found to invade the lungs (**A**) and the liver (**B**) of C57BL/6J mice without any tapeworm infection. In *T. crassiceps* infections, melanoma were also found in the lungs (**D**) and the liver (**E**), while in *M. corti* infections, they were only found in the liver (**H**), while the lungs were clear of them (**G**). Zoomed-in areas of the liver metastases (**C**, **F**, **I**).
