# Supplementary material for: Suppression of the growth and metastasis of mouse melanoma by Taenia crassiceps and Mesocestoides corti tapeworms
Source: Front Immunol. 2024 Mar 20;15:1376907. doi: 10.3389/fimmu.2024.1376907 (PMC10987685; doi:10.3389/fimmu.2024.1376907)
Supplement: Supplementary Figure 4 — Titration of MelH antigen. There was significant change in IgM binding to MelH only when the antigen concentration was increased 20x, showing that the IgM probably bound to the antigen only in a non-specific manner, after analysis with Kruskal-Willis test with Dunn’s multiple comparisons. C57BL/6J mouse sera were tested (n = 5), infected with either T. crassiceps or M. corti. [file Image_4.pdf]

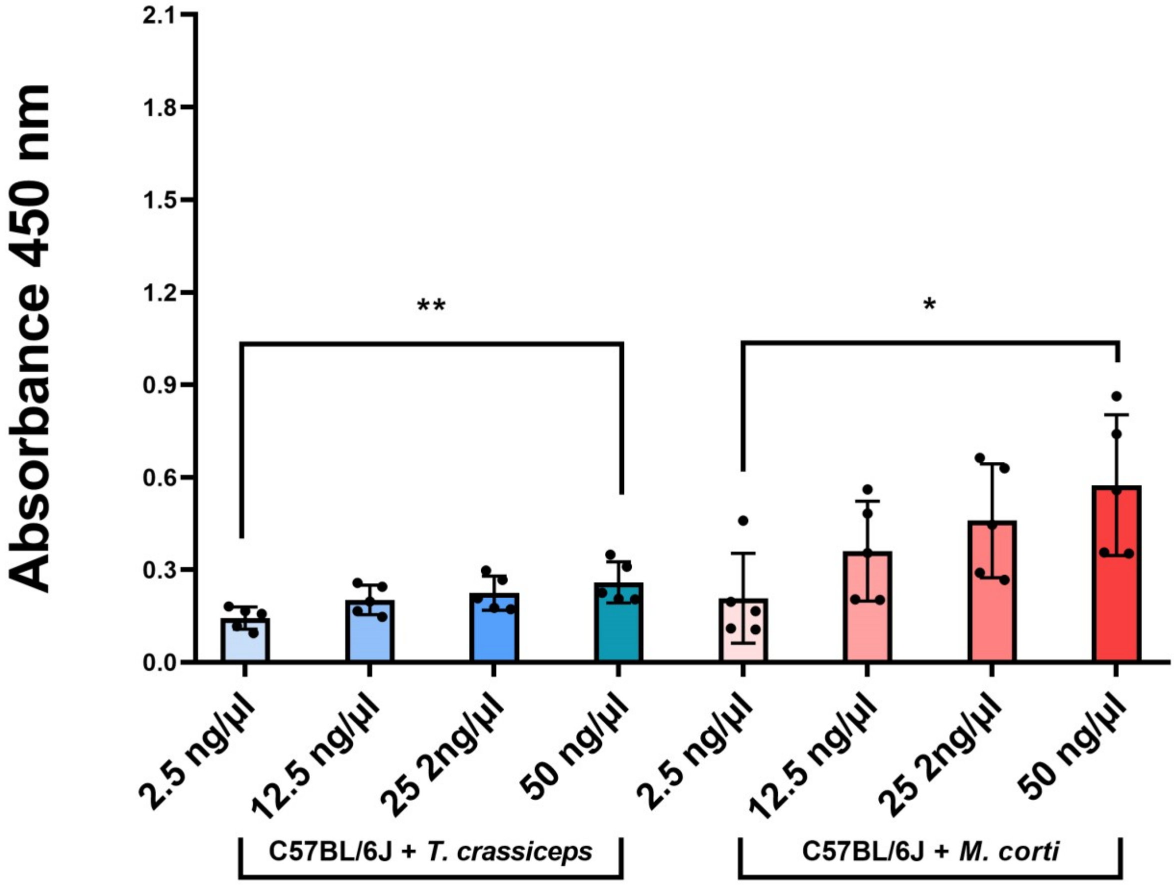

SFig. 4. Titration of MelH antigen. There was significant change in IgM binding to MelH only when the antigen concentration was increased 20x, showing that the IgM probably bound to the antigen only in a non-specific manner, after analysis with Kruskal-Willis test with Dunn's multiple comparisons. C57BL/6J mouse sera were tested (n = 5), infected with either *T. crassiceps* or *M. corti*.
