# Supplementary material for: Suppression of the growth and metastasis of mouse melanoma by Taenia crassiceps and Mesocestoides corti tapeworms
Source: Front Immunol. 2024 Mar 20;15:1376907. doi: 10.3389/fimmu.2024.1376907 (PMC10987685; doi:10.3389/fimmu.2024.1376907)
Supplement: Supplementary Figure 5 — Antibody response to tapeworm and melanoma antigens of ICR mice infected with T. crassiceps and M. corti. Here, the mouse strains and infections are as follows: A – D ICR mice infected with T. crassiceps, E – F ICR mice infected with M. corti. Sera of all infected mice contained high levels of specific IgM (A, E) and IgG (B, F) raised against the antigens of the respective tapeworm. In contrast, they did not contain any significant amount of IgM (C, G) or IgG (D, H) specific to MelH. (n = 7) The cut-off values were determined according to Frey et al. (29). [file Image_5.pdf]

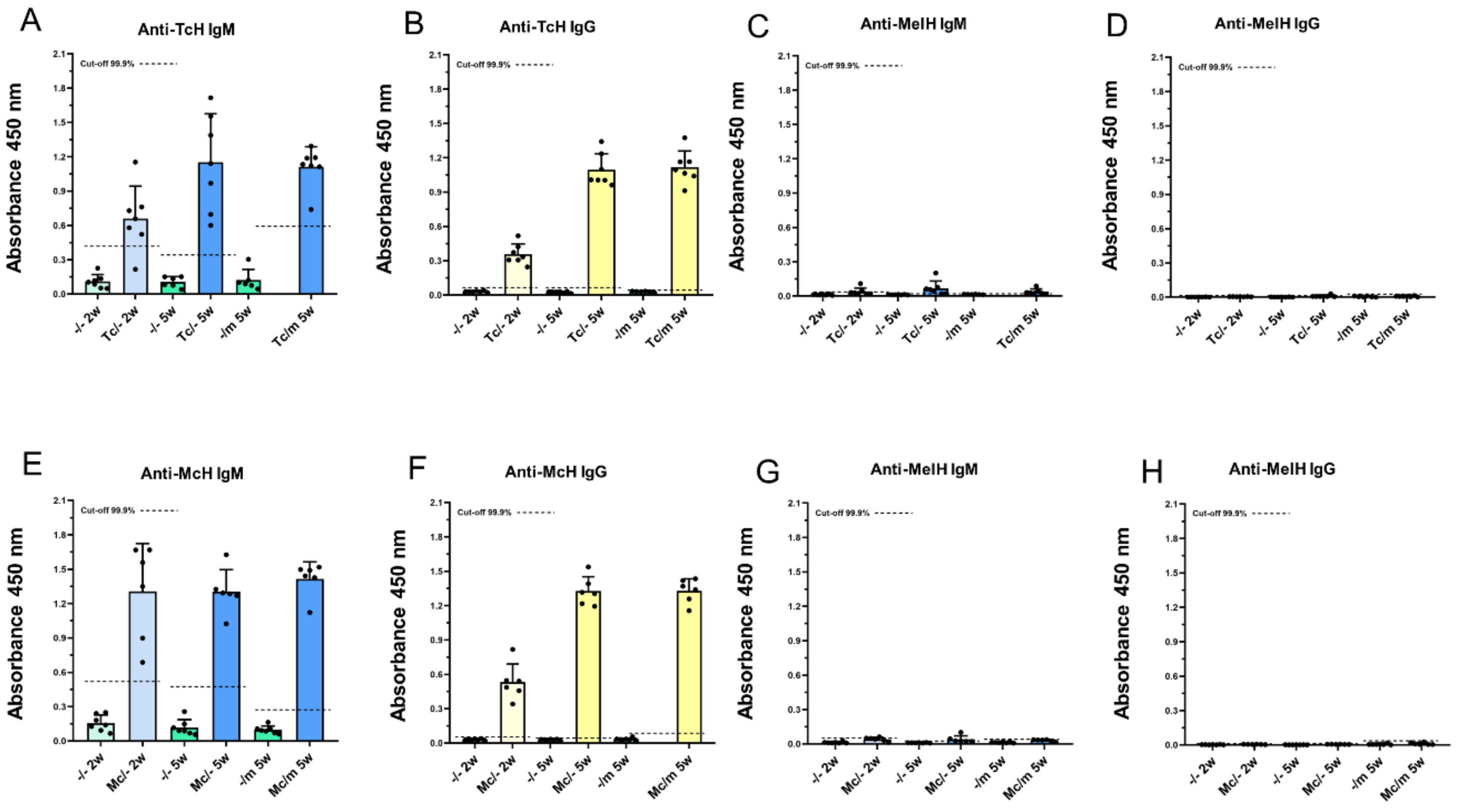

SFig. 5. Antibody response to tapeworm and melanoma antigens of ICR mice infected with *T. crassiceps* and *M. corti*. Here, the mouse strains and infections are as follows: **A – D** ICR mice infected with *T. crassiceps*, **E – F** ICR mice infected with *M. corti*. Sera of all infected mice contained high levels of specific IgM (**A**, **E**) and IgG (**B**, **F**) raised against the antigens of the respective tapeworm, while they did not contain any significant amount of IgM (**C**, **G**) or IgG (**D**, **H**) specific to MelH. (n = 7) The cut-off values were determined according to Frey et al. (1998).
