# Supplementary material for: Suppression of the growth and metastasis of mouse melanoma by Taenia crassiceps and Mesocestoides corti tapeworms
Source: Front Immunol. 2024 Mar 20;15:1376907. doi: 10.3389/fimmu.2024.1376907 (PMC10987685; doi:10.3389/fimmu.2024.1376907)
Supplement: Supplementary Figure 6 — Cytokines produced by restimulated splenocytes. An example of cytokine production by splenocytes restimulated with tapeworm homogenate or melanoma homogenate, ICR mice infected with T. crassiceps (n = 7) given as example. Values below detection limit and those above the calibration curve are omitted. – represents control, + concanavalin, MelH 1 and MelH 10 represent 1 and 10 μg/ml of melanoma cell homogenate, TcH 1 and TcH 10 then 1 and 10 μg/ml of T. crassiceps homogenate. Statistical analysis was performed with Kruskal-Willis test with Dunn’s multiple comparisons test. [file Image_6.pdf]

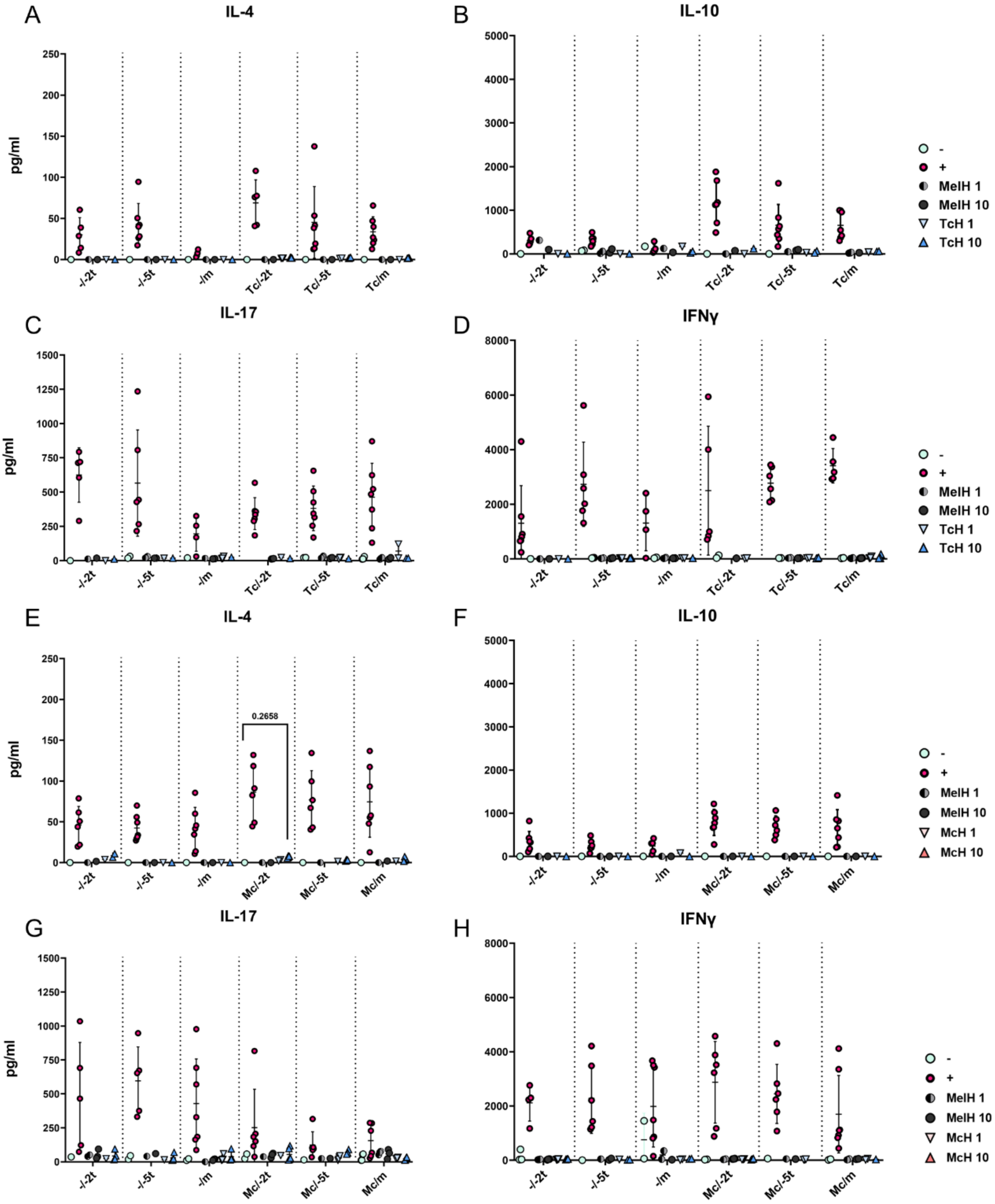
A
IL-4
B
IL-10
C
IL-17
D
IFNγ
E
IL-4
F
IL-10
G
IL-17
H
IFNγ
pg/ml
0.2658
-/-2t
-/-5t
-/m
Tc/-2t
Tc/-5t
Tc/m
Mc/-2t
Mc/-5t
Mc/m
-
+
MelH 1
MelH 10
TcH 1
TcH 10
McH 1
McH 10

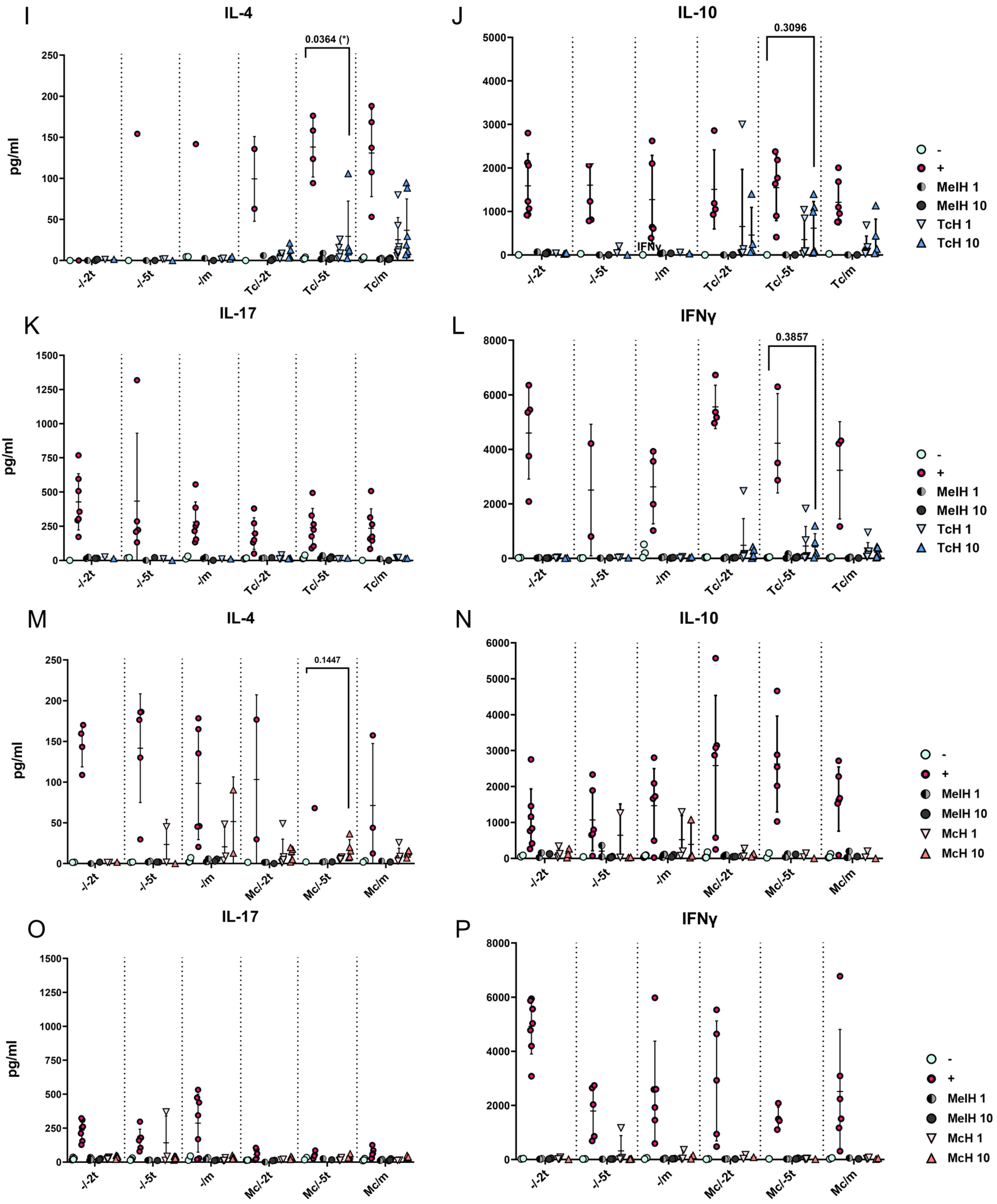

I
IL-4
0.0364 (*)
pg/ml
-/-2t
-/-5t
-/m
Tc/-2t
Tc/-5t
Tc/m
J
IL-10
0.3096
IFNγ
K
IL-17
L
IFNγ
0.3857
-
+
MelH 1
MelH 10
TcH 1
TcH 10
M
IL-4
0.1447
Mc/-2t
Mc/-5t
Mc/m
N
IL-10
O
IL-17
P
IFNγ
McH 1
McH 10

**SFig. 6. Cytokines produced by restimulated splenocytes.** Cytokine production by splenocytes restimulated with tapeworm homogenate or melanoma homogenate (n = 7). **A – D** are C57BL/6J mice infected with *T. crassiceps*, **E – H** are C57BL/6J mice infected with *M. corti*, **I – L** are ICR mice infected with *T. crassiceps* and **M – P** are ICR mice infected with *M. corti*. Values below detection limit and those above the calibration curve are omitted. – represents control, + concanavalin, MelH 1 and MelH 10 represent 1 and 10 µg/ml of melanoma cell homogenate, TcH 1 and TcH 10 then 1 and 10 µg/ml of *T. crassiceps* homogenate, McH 1 and McH 10 *M. corti* homogenate. -/- 2t and 5t are control mice of age equivalent to mice 2 or 5 weeks post tapeworm infection, -/m are mice with only melanoma, Tc or Mc/- 2t and Tc or Mc/- 5t are mice with only tapeworms, 2 or 5 weeks post infection and Tc or Mc/m are mice with both tapeworms and melanoma. Statistical analysis was performed with Kruskal-Willis test with Dunn's multiple comparisons test.
