# Supplementary material for: Suppression of the growth and metastasis of mouse melanoma by Taenia crassiceps and Mesocestoides corti tapeworms
Source: Front Immunol. 2024 Mar 20;15:1376907. doi: 10.3389/fimmu.2024.1376907 (PMC10987685; doi:10.3389/fimmu.2024.1376907)
Supplement: Supplementary file 7 [file Table_2.docx]

**Table 2.** **Weights and placement of peritoneal melanoma, a comparison of ICR and C57BL/6J mice.** ( **–** signifies no weighable tumors present, **x** signifies a mouse which died before the experiments concluded, **/** - no infection, **Tc** - *T. crassiceps*, **Mc** - *M. corti*).

| Nr. | Infection |  | Melanoma weight (g) |
| --- | --- | --- | --- |
| ICR | | | |
| 1 | / | Miniature tumors | 0.0511 |
| 2 | / | Miniature tumors | 0.0963 |
| 3 | / | Miniature tumors in fatty tissue | - |
| 4 | / | Miniature tumors | - |
| 5 | / | Miniature tumors in fatty tissue, site of injection | - |
| 6 | / | Thymus, lungs, 1 liver lobe | - |
| 7 | / | Peritoneal tumors, 1 lung lymph node | 0.2606 |
| 8 | / | - | - |
| 9 | / | Miniature tumors in peritoneal cavity, liver | - |
| 10 | / | Miniature tumors in peritoneal cavity, lung lymph nodes | - |
| 11 | / | Miniature tumors on lungs, gall bladder, mesenteric LN |  |
| 12 | / | Large amount of tumors, bloody ascites, thymus | 1.3253 |
| 13 | / | Miniature tumors in peritoneal cavity | - |
| 14 | / | Peritoneal tumors, on liver, lung lymph nodes |  |
| 1 | Tc | - | - |
| 2 | Tc | Site of injection | 0.0039 |
| 3 | Tc | Site of injection, liver tumor | - |
| 4 | Tc | - | - |
| 5 | Tc | Site of injection | - |
| 6 | Tc | - | - |
| 7 | Tc | - | - |
| 1 | Mc | - | - |
| 2 | Mc | x | x |
| 3 | Mc | Miniature tumors on liver | - |
| 4 | Mc | Site of injection | - |
| 5 | Mc | - | - |
| 6 | Mc | - | - |
| 7 | Mc | - | - |
| C57BL/6J | | | |
| 1 | / | Large tumors, bloody ascites, lung lymph nodes, site of injection | 5.0148 |
| 2 | / | x | x |
| 3 | / | Large tumors | 3.7222 |
| 4 | / | Large tumors, thymus, lung lymph nodes, liver | 4.422 |
| 5 | / | Large tumors, in lungs | 4.4247 |
| 6 | / | Large tumors, lung lymph node, site of injection | 4.3759 |
| 7 | / | x | x |
| 8 | / | Large tumors, thymus, lung lymph nodes | 4.0230 |
| 9 | / | Large tumors, lung lymph nodes | 3.8002 |
| 10 | / | Miniature tumors | 0.0168 |
| 11 | / | Miniature tumors | 0.2737 |
| 12 | / | Large tumors, bloody ascites, thymus | 3.965 |
| 13 | / | Large tumors, bloody ascites, spleen | 3.1172 |
| 14 | / | Miniature tumors | 0.0185 |
| 1 | Tc | >0.5cm tumors, miniature tumors | 0.6174 |
| 2 | Tc | Smaller tumors, site of injection | 1.1308 |
| 3 | Tc | Smaller tumors | 0.2154 |
| 4 | Tc | Site of injection | 0.2479 |
| 5 | Tc | Mesenteric lymph node | 0.087 |
| 6 | Tc | Smaller tumors | 1.5116 |
| 7 | Tc | Large tumors | 6.4172 |
| 1 | Mc | Miniature tumors | 0.0994 |
| 2 | Mc | Smaller tumors, all lung lobes affected | 2.9646 |
| 3 | Mc | Miniature tumors, mesenteric lymph node | 0.282 |
| 4 | Mc | Miniature tumors | 0.1267 |
| 5 | Mc | Miniature tumors | 0.0079 |
| 6 | Mc | Miniature tumors, mesenteric lymph node | 0.0246 |
| 7 | Mc | Miniature tumors, mesenteric lymph node | 0.448 |
